# Parents with intellectual disability reporting on factors affecting their caregiving in the wake of the COVID‐19 pandemic: A qualitative study

**DOI:** 10.1111/jar.13026

**Published:** 2022-08-05

**Authors:** Tommie Forslund, Stina Fernqvist, Helena Tegler

**Affiliations:** ^1^ SUF‐Resource Center, Region Uppsala Uppsala Sweden; ^2^ Department of Psychology Stockholm University Stockholm Sweden; ^3^ Centre for Social Work (CESAR), Department of Sociology Uppsala University Uppsala Sweden

**Keywords:** a stress‐resources perspective, adapted information, caregiving demands, coping resources, parents with intellectual disability, the COVID‐19 pandemic

## Abstract

**Background:**

Parents with intellectual disability are vulnerable to parenting stress and overwhelming life events. The COVID‐19 pandemic constitutes a potentially overwhelming event, but there is little knowledge concerning the effects on parents' caregiving. The present study aimed to fill this gap.

**Method:**

Semi‐structured interviews with 10 Swedish parents with intellectual disability were analysed using thematic analysis.

**Results:**

One broad caregiving‐related theme: increased caregiving demands and reduced resources for coping resulting in strained parent–child interactions and relationships. Four subthemes highlighted influential factors: pandemic information, professional support, social relationships and informal support, and children's school activities. Strained parent–child interactions were particularly common in the absence of adapted pandemic information, if professional and informal support were compromised, and if the parents had dealt with school‐related changes.

**Conclusions:**

Findings support contextual models of caregiving and a stress‐resources perspective, and emphasise the importance of adapted information and support to parents with intellectual disability during crises.

## 1 INTRODUCTION

Parents with intellectual disability are vulnerable to caregiving difficulties (McConnell et al., 2021), susceptible to parenting stress (Meppelder et al., 2015), and sensitive to overwhelming life events (Feldman & Aunos, 2020). The COVID‐19 pandemic constitutes a potentially overwhelming event, and persons with intellectual disability have been prone to pandemic related negative effects (Courtenay & Perera, 2020; Lunsky et al., 2022). However, there is little knowledge concerning potential effects on the caregiving of parents with intellectual disability. The present study addresses this knowledge gap.

## 2 PARENTS WITH INTELLECTUAL DISABILITY AND MULTIFACTORIAL INFLUENCES ON CAREGIVING

Parents with intellectual disability and their children constitute a joint high‐risk group. In this high‐risk group, the parents are at risk for caregiving difficulties (Wade et al., 2008), the children for behaviour problems (Collings & Llewellyn, 2012), and the families for child out‐of‐home placements (McConnell et al., 2021). Similar to parents without intellectual disability, the caregiving of parents with intellectual disability depends on factors pertaining to both the parents, the children, and the broader social context (Feldman & Aunos, 2020). However, contextually based risk factors are overrepresented, including but not limited to, socioeconomic disadvantage (Fernqvist, 2015), single parenthood (Walton‐Allen & Feldman, 1991), limited social support (Wade et al., 2015) and mental health problems (Hartley & MacLean Jr, 2009). The children are also at risk for genetically based vulnerabilities (McConnell et al., 2003). Such factors explain a notable amount of the variation in caregiving and child development among these families (Schuengel et al., 2017).

While the reasons for caregiving difficulties are multifactorial, parents with intellectual disability and their children are vulnerable and often need support (Wade et al., 2008). Parenting stress is very common (Aunos et al., 2008; Feldman et al., 2002), and theoretical models on caregiving and intellectual disability emphasise a stress‐resources perspective (Meppelder et al., 2015) and a sensitivity to overwhelming life events (Feldman & Aunos, 2020). As captured by transactional models, stress often results from an imbalance between demands and coping resources (Lazarus & Folkman, 1984). Applied to parents with intellectual disability, these parents often face increased caregiving demands (e.g., children with special needs) and limitations in internal (e.g., adaptive behaviour) and external (e.g., social support) resources for coping (Feldman et al., 1997). Parents with intellectual disability may thus be prone to elevated baseline levels of parenting stress, whereby significant events are at risk for becoming overwhelming.

### 2.1 Pandemic effects on persons with intellectual disability and factors influencing caregiving

The COVID‐19‐pandemic has had pervasive effects, particularly for already vulnerable individuals (Noor & Islam, 2020). Persons with intellectual disability often belong to COVID‐19 risk‐groups, and highly elevated infection and mortality rates have been reported (Gleason et al., 2021). Individuals with a history of mental illness have also been at an elevated risk for psychological problems (e.g., McCracken et al., 2020), and persons with intellectual disability have been prone to mental health problems during the pandemic (Linehan et al., 2022; Lunsky et al., 2022). The pandemic and its restrictions have also had adverse effects on occupation and income (Kawohl & Nordt, 2020), social support (Grey et al., 2020), and professional support and health care (e.g., Negrini et al., 2020). Persons with intellectual disability often need health care and depend in part on informal and professional support, but have faced reduced and delayed support, staff changes, and digitalization of support (Courtenay & Perera, 2020; Lunsky et al., 2022; Willner et al., 2020; Zaagsma et al., 2020). There has also been a scarcity of adapted information, entailing a risk for limited understanding, increased worrying, and diminished opportunities for coping (Chadwick et al., 2022; Lake et al., 2021). In Sweden, there was a notable reduction in professional support to persons with intellectual disability during the first stages of the pandemic, and the government was criticised for delays in ensuring adapted information (FUB, 2021). A Swedish interview‐based study with persons with intellectual disability also documented highly adverse pandemic‐related effects, including loneliness, isolation and psychological problems (Tideman et al., 2021).

The COVID‐19‐pandemic has constituted a potentially overwhelming event for both parents and children through closures of day care and homeschooling, reduced contact with friends and family, and disruptions to activities and routines (Lee et al., 2021; Thorell et al., 2022). It has, therefore, been argued that the pandemic may have been particularly challenging for family well‐being, and especially so for families who are vulnerable due to socioeconomic adversity, caregiver mental health problems or children having special needs (Prime et al., 2020). Pandemic research on caregiving is still relatively scarce, but studies have reported increased caregiving responsibilities, parenting stress and strains on parental capacities (Calvano et al., 2021; Lee et al., 2021; Roos et al., 2021). Pandemic research on risk and resilience has also emphasised bidirectional influences between parents and children, and linked lower‐quality parenting to pre‐pandemic mental health problems and limited social support, the magnitude of pandemic‐related stressors, and caregiver mental health problems and unmet childcare needs during the pandemic (Beach et al., 2021; Jones et al., 2022). Most research thus far has been conducted with families with relatively high socioeconomic status, and there is as of yet no research on parents with intellectual disability. However, research on the pandemic effects for caregivers whose children have intellectual disability have reported increased caregiving responsibilities, reduced professional and social support, caregiving stress and extremely high levels of mental health problems (Patel et al., 2021; Rogers et al., 2021; Willner et al., 2020).

### 2.2 Aim of the present study

The findings reviewed above suggest that pandemic effects on persons with intellectual disability may extend to the caregiving of parents with intellectual disability. There is, however, little research on this important matter. This is unfortunate, since such knowledge may inform support during future significant societal events. In addition, the majority of research concerning individuals with intellectual disability during the COVID‐19 pandemic stem from reports by secondary sources (e.g., support staff, caregivers). There is thus a need for first‐hand research that gives voice to these individuals' experiences (for a discussion, see Arvidssson et al., 2016). We, therefore, examined how Swedish parents with intellectual disability have experienced the pandemic, with a focus on caregiving and influential factors.

## 3 METHODS

We used semi‐structured interviews and qualitative analyses. The Swedish Ethical Review Authority approved the study (2021‐00436).

### 3.1 Participants

Parental inclusion criteria were (1) mild intellectual disability, (2) active parenthood, and (3) ability to participate without an interpreter. We included parents whose children were between preschool and high school age. We also included both parents whose children lived with them full time, separated parents whose children lived with them part‐time, and parents whose children were in out‐of‐home care but in regular contact. However, we did not include parents whose children were all adults, living independently, and presumably not requiring as much active guidance as younger children.

We recruited parents from several Swedish regions, through practitioners working with parents with cognitive difficulties (e.g., intellectual disability, ADHD, ASD). In Sweden, being diagnosed with intellectual disability is a prerequisite for support and service pursuant to the entitlement law ‘The Swedish Act concerning Support and Service for Persons with Certain Functional Impairments’. As such, the diagnosis is often known to professionals. The practitioners gave adapted verbal and written information about the study to the parents and asked them about their interest in participating. With the parents' consent, the practitioners forwarded their contact information to the researchers, who gave further information about participation over the telephone.

The final sample consisted of 10 parents (nine mothers) between 23 and 54 years of age (*M* age = 40.8). To maintain the confidentiality of the families, the sample is described generally, rather than in a table with demographic data for the families. Eight parents reported additional, coexisting cognitive difficulties (e.g., ADHD, ASD, Dyslexia), and six parents reported coexisting psychiatric problems (e.g., depressive and/or affective disorders). One parent reported having contracted COVID‐19. Nine parents lived in rental apartments, five had work/daily activities, four identified as single, and four were in a relationship with their child's/children's other biological parent. In total, the parents had 19 children (11 boys) between 6 and 27 years of age (*M* age = 15.8). The parents had between one and four children, with six of the parents having two children. Ten children were diagnosed with or undergoing assessment for cognitive difficulties (e.g., intellectual disability, ASD, ADHD).

Ten children lived primarily with the interviewed parent and one lived primarily with the other parent. Four children were in out‐of‐home care and four were adults who lived independently. Some parents had both children living with them full‐time and children in out‐of‐home care. In total, eight parents had children who lived with them full time, six of whom had one child living with them, and the others two children. Of the remaining two parents, one had had both children placed in out‐of‐home care during the pandemic (and were in regular contact), and the other (separated) parent's child stayed with her every other weekend.

### 3.2 Procedures/data collection

We developed a study‐specific interview manual (available upon request) based on research regarding factors influencing caregiving among parents with intellectual disability (e.g., Feldman & Aunos, 2020), a group discussion with practitioners concerning perceived effects on parents with intellectual disability, and pilot testing with a parent with intellectual disability. This resulted in a broad interview manual with 20 questions, divided into three sections, concerning the effects of the pandemic on the parents (e.g., psychological health, occupation, support), their children (e.g., extracurricular activities, school, peer relationships) and parent–child interactions and relationships (e.g., time together, joint activities, conflicts). Finally, we asked four closing questions (e.g., recommendations for future pandemics). Each interview was adapted to facilitate understanding and responding through concrete wording of questions, follow‐up probes, and repeating and rephrasing questions if needed. We introduced each question with clarifying examples of what was meant.

Eight parents chose to be interviewed at a neutral place (e.g., a habilitation centre) and two parents chose video calls (due to fear of contracting COVID‐19). One parent wanted to have a support person present. Data collection began with giving information about participating in research (e.g., anonymity, confidentiality, voluntary participation). All parents gave (written) informed consent to participating in the study. The data collection then proceeded with background questions (e.g., occupation, number of children, diagnoses). The interviews concerning the COVID‐19 pandemic were then conducted and audio‐recorded with parents written consent using a voice‐recorder. The interviews were conducted between May and August 2021, ranged between 36 and 107 min (*M* = 67 min), and were transcribed verbatim. All parents received a gift as a token of appreciation for participating (equivalent to approximately 9 EUR).

### 3.3 Data analysis

We coded the interviews using thematic analysis (e.g., Braun & Clarke, 2006). We familiarised ourselves with the data by reading the transcripts, then generated initial codes and searched for themes independently. We collated codes using NVivo (V.12; QSR International, 2018), discussed themes until agreement, and then named and defined these.

## 4 RESULTS

Parents with intellectual disability are a heterogeneous group, and this manifested in a notable variation in how the parents in our study fared during the pandemic. However, the majority of parents experienced a substantial impact of the pandemic and there were overarching commonalities. One broad caregiving‐related theme became evident: *Increased caregiving demands and reduced resources for coping resulting in strained parent–child interactions and relationships*. All parents described increased caregiving demands, though the reasons, magnitude and continuity varied to some extent. Also, most parents described reduced coping resources, and strained parent–child interactions and relationships were common. At the same time, the parents showed variation in coping ability. Whereas some seemed to handle the increased caregiving demands satisfactorily, others struggled.

We found four subthemes emphasising factors influencing caregiving demands and coping resources: *pandemic information*, *social relationships and informal support*, *professional support* and *children's school activities*. Parents with intellectual disability are in most respects like parents without intellectual disability, as exemplified by the importance of social support as an external coping resource, and adaptations to children's school activities resulting in increased caregiving demands. However, parents with intellectual disability also differ from parents without intellectual disability through their intellectual limitations, which may result in reduced internal coping resources. This was notable in universal reports of difficulties with general pandemic information, and common difficulties with problem solving in challenging caregiving situations. Parents with intellectual disability thus tend to be more vulnerable than parents without intellectual disability, and the importance of professional support as an external coping resource was evident. The increased vulnerability may also have manifested in more pronounced effects of factors that also affected parents without intellectual disability, such as reduced social support and adaptations to children's school activities. We first present the main caregiving‐related theme and subthemes, and then discuss the findings in relation to a stress‐resources perspective.

### 4.1 Increased caregiving demands and reduced resources for coping resulting in strained parent–child interactions and relationships

Caregiving demands increased due to effects related to the children, who faced school‐related changes, cancelled leisure activities, and reduced contact with peers and family. This often led to children becoming moody and reactive, and was particularly prominent for children with special needs who reacted strongly to changes in routines and support. As such, children spent more time at home and required more guidance from their parents. While the parents regarded more time together as positive, the added work for the parents in guiding and activating their children, and the increased volume of household work (e.g. groceries, cooking, cleaning), was taxing for most and overwhelming for some:

You try to come up with activities…, play music, dance, build forts… But that's the thing, you must put everything away afterwards. And when it keeps mounting up, eventually you don't have any energy left for the kids. When they go to bed you can't do it, instead you just sit on the couch or go to bed because you are so wiped out.

Most parents also mentioned parent–child activities that they could not do (e.g., go to the public indoor swimming pool or to flea markets). That meant increased pressure to come up with new joint activities, and some parents mentioned outdoor activities, board games, cooking, baking or doing handicraft. Other parents seemed to have difficulties being inventive, and these parents tended to note that their children became moody and withdrawn:

She became very quiet… I don't know, it seemed like she became very kind of depressed, sad, pondering, feeling bad.

The COVID‐19 pandemic also led to hampered parent and child moods. Not surprisingly, it led to concerns about life and death. While some parents merely felt uneasy, notable worry was common and some even reported mortal fear. Such worries included their children, several of whom also worried about their parents. Hampered parent moods further reduced coping resources, and child negative moods increased caregiving demands, straining parent–child interactions, contributing to conflicts and reducing relationship satisfaction. One parent stated that ‘we have become more aggressive toward each other’ and several parents noted they had less energy and patience for their children and were more easily annoyed. Likewise, several parents noted that their children became angry or sad and depressed:

My son became much more aggressive, angry, throwing things, acting out. I think it was because he…well, did not have an outlet as he usually does.

The amount of time she spends on the phone. She quickly gets very angry and tells me to shut up, to go to hell.

While most parents noted relationship strains, it sometimes had drastic consequences. One parent eventually felt compelled to place her children in out‐of‐home care, due to the pandemic's impact on her wellbeing and caregiving:

That's when I said “enough, the children must move”, cause I do not want them to suffer (…) And if I feel like… no, I can't do the cooking, ask my child “can you cook?”, then it's gone too far, then something must change (…). I'd tried with support for so long, so I had to do the biggest, most difficult thing.

While the pandemic had substantial effects on the parents, there was certainly variability in demands, coping, and strains. We elaborate on this heterogeneity through the subthemes, addressing influential factors.

### 4.2 Sub theme 1:1 pandemic information

Difficulties with abstract information is a hallmark of intellectual disability, and the parents universally expressed difficulties understanding governmental information and news reports about the pandemic. The parents also had difficulties finding adapted information, and they perceived the governmental information and news reports as fragmentary, contradictory, and frightening.

Will soldiers come? Will people stop us in the streets? Will people nail our doors shut? What's happening? When I watched the news that was what I saw, in other countries. (…) Then it was said that bats spread the virus … I've seen two bats, could they be here, on my balcony? What's going on? I became more and more afraid.

Some parents eventually acquired adapted information, from friends/family or professionals, and others showed inventiveness in acquiring it. For instance, one parent contacted an organisation for people with intellectual disability, and another watched a news program for children. These parents tended to exhibit general adaptability:

We go to two stores for groceries, where we've figured out the best times to go. I run in one direction, and my husband in another. We have two shopping lists. “Fast in, fast out.”

Other parents had lingering difficulties acquiring adapted information and understanding the pandemic, and this seemed to take a toll on their well‐being and general functioning (e.g., sleep problems, anxiety). Continued difficulties with pandemic understanding also seemed to co‐occur with their ability to cope with caregiving challenges, such as finding new joint activities and guiding and supporting their children. More specifically, these parents gave responses that, while reducing their own anxiety, seemed categorical, refraining from activities that parents have often found appropriate and increasing isolation.

We [the informant and another parent] used to do many things together with our kids. (…) We used to go playgrounds, and had lots of fun. But during the pandemic, we could not meet. He was like “but we can be outdoors, as long as we keep a distance”… but then my fear came, it stopped me a whole lot.

In summary, continued difficulties with pandemic information decreased the ability to cope, contributing to impoverished joint activities and increased family isolation. The role of formal and informal support in acquiring understanding relates this subtheme to the other subthemes.

### 4.3 Sub theme 1:2 professional support

Continuous compensatory and caregiving‐based support, from familiar professionals given in the home, is important for parents with intellectual disability. However, the pandemic resulted in reduced and changed support for many parents. Contact persons, companions, and housing support were for instance paused for months, which left the parents alone in dealing with everyday household tasks (e.g., cleaning, shopping, cooking, paying bills). The withdrawal of support was frustrating and affected the parents' self‐perception as capable grown‐ups and parents:

I called the head office and said ‘what the h‐ should I do? This bill has to be paid, how do I do it? I need them [the contact persons]. Well, we then got to meet outside my house, in a snowstorm and… it didn't work.

For some parents, reduced support caused disastrous consequences. One mother described how she, instead of being a happy and lively mother, became withdrawn and felt depressed.

I usually say that my housing support makes me bloom. Now, I'm like a wilted rose, because I do not get the support I need.

When everyday routines and housing support disappeared, chaos appeared, and video calls were an inadequate substitute:

Everything just fell apart. The supporter could no longer visit, you could get video calls, but that was not the same. You weren't able to have people at your house anymore.

Another important loss was the municipality‐arranged Family Centers for parents with cognitive difficulties and their children, offering a community, enjoyable activities, and support from well‐known staff.

I have had phone calls with the family support, but that's not been very good, because both my daughter and I should get support, but they were just with me, my daughter did not get to receive any support.

Many parents and children needed healthcare or psychiatry, but it was often paused or rescheduled to video calls. Digital platforms were troublesome, and several parents cancelled or postponed contacts since they did not know how to connect or felt unsure interpreting emotional expressions. Since an intellectual disability may entail special communicative demands (e.g., wanting to read lips and facial expressions), it is not surprising that digital contact was challenging.

I have cancelled many [meetings], emotional expressions are really difficult for me, I need to see if you're happy, sad or angry in order to understand.

Paused interventions also meant difficulties in getting child health care and medication. One parent demonstrated adaptive functioning as she went to the child‐ and adolescent psychiatry and argued for her child's needs.

I had a quarrel with a receptionist: ‘The doctor did not get back to me and I have a kid at home without medication. What should I do? Tell me! I won't leave before you tell me’.

In summary, the parents expressed difficulties with various types of professional support, for themselves as well as their children, resulting in increased caregiving demands and reduced external resources for coping.

### 4.4 Sub theme 1:3 social relationships and informal support

The reduced or absent opportunities to see loved ones was a drastic consequence for the parents and their children that was associated with sadness and isolation. Many children longed for friends and family and kept asking when they could meet, and several parents mentioned conflicts from their children not understanding (or wanting to follow) the restrictions. Apart from the social loss, the pandemic also resulted in reflections about one's responsibility and fears of infecting loved ones:

My grandparents, I hardly wanted to meet them, or my great grandmother. I'm terrified of infecting people. You don't want to have on your conscience that you accidentally…caused someone to die, because of you, who otherwise could've locked yourself up in your home.

Reduced professional support made informal support more significant for some parents, and one parent's family helped her paying the bills when the contact person could not visit. Physical distancing often led to reduced social support. In the private sphere, some parents noted reduced support from their family who, for instance, previously helped their child get ready for school or helped pick up the child after school. Reduced informal (and professional) support may have had a particular impact on the parents who were single. More specifically, the parents who were single seemed to fare worse during the pandemic than those who had a partner, all of whom had been in a stable relationship for years. While the parents' responses did not clearly emphasise the importance of their partners for their coping, a partner conceivably meant having someone to share the load with, and emotional and instrumental support. Effects on social support and social relationships were also notable during work and daily activities, through decreased work opportunities and fewer coworkers being present. This was strenuous for the parents, several of whom regarded their coworkers as close friends. Indeed, some parents used to talk with their coworkers about parenting and gave each other advice. The reduced social contact thus took a toll on the parents' well‐being:

When one doesn't have that much social contact after work and friends, that's the spice [of life], to have a coworker to talk with. So I became a bit depressed.

The shift to digital encounters was challenging both privately and during work and daily activities. While digital contact was unproblematic for some, several parents viewed it as an inadequate substitute that contributed to less contact with friends and family.

It has always felt better to… meet someone and talk. Sure, I can be on the phone for hours, but it's not the same, not the same togetherness.

In summary, most parents experienced negative effects on social relationships and informal (practical, emotional) support, resulting in increased caregiving demands and reduced external coping resources.

### 4.5 Sub theme 1:4 children's school activities

The pandemic had pervasive effects on the children's school activities. They recurrently had substitute teachers (or were sent home) when teachers were sick, and many children had teacher‐led distance education. The children often struggled to keep up, since they required monitoring and the opportunity to ask questions. Distance education thus increased caregiving demands, and the parents found this stressful since they could not help their children with schoolwork:

There were many times [where the son said], ‘I need help, I don't understand this’. But I don't have the knowledge. I couldn't help him.

It was sometimes uncertain from one day to the next if there would be in‐school activities, and this caused particular problems for children with cognitive challenges. Some children even refused to go to school, and one parent described how her daughter isolated herself, fell behind, and did not receive passing grades. Since school is important for children's social life, the pandemic also had problematic effects on peer relationships. One parent described how her child lost all friends. Such problematic social effects made children sad and moody, further increasing caregiving demands. Some parents found this difficult to cope with, and one mother, unable to help her child with peer and school problems, described how they used to comfort eat together.

One mother interpreted the doctor as confirming that her daughter belonged to a COVID‐19 risk‐group. She thus kept her daughter at home for months, and the daughter missed lots of schoolwork before the school summoned the mother:

It was as if I'd lied about it all [the daughter's risk‐status], but I didn't. I'd misunderstood the doctor, because he wasn't clear enough with me.

Well‐functioning communication between parents and teachers is important. However, the parents' difficulties with written information posed problems as such communication increased. One mother described how her son's previous teacher sent text messages to make sure she understood. However, the child switched teachers during the pandemic, and the new teacher did not facilitate her understanding. Instead, the school contacted social services, suspecting the parent of neglect. The mother asked for a meeting but the teacher declined:

The teacher said, “I think you're like this as a mother, and these faults are not acceptable”. Then I said to the teacher ‘you don't understand people with disabilities, and if you'd wanted to listen, we could have solved this’.

In summary, interacting with schools is an established source of stress for parents with intellectual disability, and the pandemic increased such stress and influenced the caregivers both indirectly (through effects on their children) and directly (through effects on themselves).

## 5 DISCUSSION

This study examined how parents with intellectual disability fared during the COVID‐19‐pandemic and factors influencing their caregiving. Strains on parent–child interactions were common, and some parents experienced a caregiving crisis. This is in line with research stressing that parents with intellectual disability are especially vulnerable (Wade et al., 2008). Influential factors, such as accessibility of adapted information, and professional and social support, also corroborate theory and research on the importance of contextual factors for the caregiving of parents with intellectual disability (Feldman & Aunos, 2020). The influence of these factors during the pandemic is not unique for parents with intellectual disability (Jones et al., 2022; Lee et al., 2021; Roos et al., 2021; Thorell et al., 2022). However, parents with intellectual disability may have been more heavily impacted due to lower levels of internal and external coping resources (Feldman et al., 1997). They may also have been more heavily impacted due to high baseline levels of caregiving demands and stress, similar to parents of children with special needs, not least due to their need for formal and informal support in order to cope with parental responsibilities (Beach et al., 2021; Patel et al., 2021; Rogers et al., 2021; Willner et al., 2020). At the same time, some of the parents showed evidence of coping resources and fared comparatively well, highlighting heterogeneity and lending credence to a stress‐resources perspective (Meppelder et al., 2015).

### 5.1 Stress and resources

The pandemic's broad effects resulted in increased caregiving demands, reduced coping resources, and increased parenting stress, in line with stress‐resources perspectives (Meppelder et al., 2015; Prime et al., 2020). This was in part due to effects on the children, who were moodier and required more guidance, attesting to bidirectional effects between children and caregivers during the pandemic (Jones et al., 2022). It was also in part due to reduced social and professional support, which further increased caregiving demands. It is important to note that the majority of the parents in the present study had children with cognitive challenges, and it is thus likely that the pandemic exacerbated already high caregiving demands and parenting stress. Highly adverse effects of the pandemic on parenting stress and mental health have indeed been found among caregivers to children with intellectual disabilities, who also faced increased demands and reduced support (Patel et al., 2021; Rogers et al., 2021; Willner et al., 2020). However, the parents' internal and external coping resources varied, contributing to different levels of stress and caregiving‐related strain.

Regarding internal resources, some parents seemed more flexible and creative in dealing with challenges and getting their own and their children's needs met. They found new parent–child activities, circumvented problems with their children's education, and engaged with authorities when support was threatened. The importance of such flexibility in problem solving has been emphasised in relation to family resilience during the pandemic (Prime et al., 2020), and may in part attest to higher levels of adaptive behaviour, which includes both adjusting to the environment and adjusting the environment to one's own way of functioning (Granlund & Göransson, 2019). Several parents, however, seemed to have problems with flexible adaptation, as exemplified by difficulties substituting parent–child activities. Such difficulties may in part be due to the intellectual and adaptive limitations, which may reduce the ability for problem solving in new situations. It is important to note that the majority of the parents reported pre‐existing mental health problems, and some parents reported that their mental health was negatively impacted by the pandemic. Low pre‐pandemic well‐being and mental health problems during the pandemic has been associated with lower‐quality caregiving among parents in general (Jones et al., 2022; Roos et al., 2021). Mental health problems may thus have further reduced internal resources for coping with the caregiving demands.

In regards to external resources, some parents highlighted the importance of their social network while others emphasised professional support. This is in line with research stressing the importance of informal and professional support for parents with intellectual disability (e.g., Sigurjónsdóttir & Traustadóttir, 2010; Wade et al., 2015). However, most parents in the current study reported reduced support during the pandemic, similar to parents in general (Lee et al., 2021; Roos et al., 2021). The Swedish crisis preparation has been termed insufficient, and persons with intellectual disability are characterised as having had to carry a disproportionate burden (Tideman et al., 2021). Our findings extend this conclusion to parents with intellectual disability. Professional support is often crucial for parents with intellectual disability, and its maintenance during challenging events is thus imperative. It is notable that Sweden, unlike many other countries, never went into a full lockdown, implying that societal functions were still accessible. Nonetheless, the parents in our sample reported highly negative pandemic effects. The negative effects may therefore generalise to parents with intellectual disability worldwide, and could conceivably be even more pronounced in countries enforcing tighter restrictions.

Another contextual factor pertains to the inaccessibility of adapted pandemic information. This is in line with reports from other countries (Chadwick et al., 2022; Embregts et al., 2020; Navas et al., 2021) and attests to unequal access to information. In fact, it took several months after the COVID‐19 outbreak before adapted information was available in Sweden (FUB, 2021). Our findings indicate that this has had ramifications for the caregiving of parents with intellectual disability, hindering these parents' ability to adapt, make informed decisions, and care for their children.

### 5.2 Strengths and limitations

The present study had limitations that present opportunities for future research. There were, for instance, no parents with infants and few parents with pre‐schoolers, and thus a reduced focus on becoming a new parent with intellectual disability during a crisis. A second limitation is that all participants lived in an affluent welfare state, with a low degree of social stratification and well‐functioning and equal social support systems. One could argue that severe marginalisation would therefore be difficult to find. A third limitation is that the overrepresentation of persons with intellectual disability in COVID‐19 risk groups, and the digital divide, may have rendered some parents unwilling to participate in physical meetings, and unable to participate in digital meetings. The study's strengths included the interview‐based design, which enabled a rare first‐person perspective from parents with intellectual disability (see also Arvidssson et al., 2016). This is, to the best of our knowledge, the first study concerning how parents with intellectual disability have experienced the pandemic. Our rich interview manual also ensured depth and breadth of information.

### 5.3 Conclusion and clinical implications

Our results corroborate research stressing that persons with intellectual disability, including parents with intellectual disability, have an increased risk for the effects of the COVID‐19 pandemic (WHO, 2020). Our findings also attest to the importance of contextual factors such as social and professional support and adapted information. This is in line with the WHO (2020) and UN (2020), who emphasise the importance of a disability perspective and adapted information to ensure equality of support and healthcare.

Our findings indicate a failure in supporting parents with intellectual disability and their children in Sweden during the COVID‐19 pandemic. This failure points to structural issues. Indeed, previous reports have highlighted substantial divergence between Swedish municipalities in handling support to persons with intellectual disability during the pandemic (Tideman et al., 2021). This may in part be due to the decentralisation of the Swedish welfare state and municipal self‐government. Preparation for similar future crises, including ensuring adapted information and professional support, may require stronger efforts on a national level and pertinent authorities to safeguard the needs of these parents and their children.

The findings also emphasise the importance of professionals being particularly attentive to the needs of these families during taxing life events. Indeed, it has been observed that crises such as illnesses, or a pandemic, could be “involved and interrelated in determining parenting and child and family outcomes” for these families (Feldman & Aunos, 2020, p.174). Preventative support may both reduce caregiving demands and increase internal and external coping resources, resulting in lowered baseline levels of stress and increased resiliency to stressful events.

## FUNDING INFORMATION

This work was supported by a grant from Nära vård och hälsa – Region Uppsala (Grant APCF‐942035) awarded to Ulrika Löfkvist.

## CONFLICT OF INTEREST

The authors declare that they have no conflicts of interest.

## Data Availability

Data are constituted by transcripts from interviews with parents with intellectual disability. The parents shared personal often difficult and sensitive experiences concerning effects on themselves, their children and parent‐child interactions during the Covid‐19 pandemic. The in‐depth interviews, which on average lasted about an hour per respondent, also yielded rich personal information that (although anonymized) could potentially be used to identify participants if shared. To preserve the respondents confidentiality, we therefore do not want to share the data.
